# Astronomical apology for fractal analysis: spectroscopy's place in the cognitive neurosciences

**DOI:** 10.3389/fncom.2014.00016

**Published:** 2014-02-25

**Authors:** Damian G. Kelty-Stephen

**Affiliations:** Department of Psychology, Grinnell CollegeGrinnell, IA, USA

**Keywords:** spectroscopy, fractal, power law, time series, molecular cloud, stars, perception, perception-action

Fractal structure offers new leverage for understanding cognition (Dixon et al., 2012; Kelty-Stephen and Dixon, 2012, 2013). A minority in neuroscience feels very strongly about this point, finding it either crucial (Friston et al., 2012; Van Orden et al., 2012) or patently absurd (e.g., Wagenmakers et al., 2012). The majority remain understandably mystified or bored by opaque math and ponderous debate. I propose to re-present the point through analogy to a field far removed from neuroscience, namely, astronomy, in the hopes of making the common threads clearer and less threatening. One field gazes deep into the brain; the other gazes up and away from anything on Earth. However, both kinds of scientists seek physicochemical accounts of comparably high-dimensional systems (Mesulam, 2008). They must take imperfect measurements and use elegant strategies to probe these measurements for what is not plainly obvious to the naked eye.

Fractal structure (or its absence) and its implication in cognition grows rather inoffensively out of spectral methods (i.e., “spectroscopy”) that elevated astronomy from guesswork to extremely sophisticated inquiry. The comparison of 20 years of neuroscience exploring fractal structure in cognition (e.g., Gilden et al., 1995) to 200 years of spectroscopy in astronomy is humblingly instructive (see Hearnshaw, 2010). Far from undermining physicochemical accounts of the heavens, since its recognition in astronomy (e.g., de Vaucouleurs, 1970; Mandelbrot, 1977), fractal structure has supported physicochemical accounts of star formation in ways non-fractal models could not (e.g., Larson, 2005). Comparing our 20 years with astronomy's 200, I am prepared not to live to see the fruition of similar attempts in neuroscience. I hope only to illustrate that neuroscience might learn a lot from astronomy's cosmopolitan views of spectroscopy.

We forget easily that modern astronomy was not always the scientific success we know today. Despite unresolved questions, we are awash in precise physical and chemical information about 10^11^ stars living for billions of years in each of 20^11^ galaxies (Geach, 2011; Tolstoy, 2011). Roughly 180 years ago, Comte (1835) predicted that we would never know the physicochemical details of the heavens. Astronomy was only as good as telescopes with the strongest magnification, and astronomy would never be more than guesswork projected into kinematics of these magnified dots and smears. Comte's words reflected an ignorance of the initial evidence from a new method called “spectroscopy.” And it was the subsequent development of spectroscopy that allowed astronomers to bury Comte's disparaging assessment.

What Comte didn't know about spectroscopy was that astronomical measures of celestial dots and smears carry richly patterned optical information (e.g., Fraunhofer, 1817). The full spectrum of electromagnetic radiation reached Earth only incompletely. Between star and telescope lay rich molecular clouds of dust and gas. Decomposing this radiation into a spectrum of oscillations at different scales revealed the composition of the molecular clouds because specific configurations of electrons absorbed and emitted light from specific ranges of the electromagnetic spectrum. For instance, Lockyer (1869) and Janssen (1869) identified the element later known as helium based on its absorbing and emitting light waves of length 587.6 nanometers—or, equivalently, light waves oscillating at a frequency of 5.1 × 10^14^ Hz. Specific elements composing the universe absorbed energy at specific scales of space and time. Here was the key to the universe's composition and to quashing Comte's prophecy of ignorance.

Spectroscopy denotes the broad class of analyses depicting how an observable's distribution over a wide range of measurement scales. Different kinds of spectra entail different sorts of axis labels. “Power” spectra plot oscillatory power (i.e., amplitude squared) against oscillatory wavelength or, inversely, frequency. “Energy” and “mass” spectra plot quantity across spatial scales. Scientists care about spectroscopy because, as with light through celestial molecular clouds, the distribution of observables varies with scale, and this relationship usually provides insights into the processes underlying phenomena we care about. Sometimes these processes exhibit selective response to characteristic scales, as in helium's emission spectra. Other measurements exhibit response over a continuous range of scales, and this response can increase or decrease with scale. Fractal structure is nothing but an extremely specific example of this latter case, namely, a spectrum exhibiting power-law (and thus scale-invariant) growth or decay across scales. Here we encounter a rather large fact that often goes unmentioned in the debates: There are truly no “fractal analyses”—only fractal or non-fractal patterns revealed by spectroscopic methods.

Neuroscience has a fondness for characteristic scales. For instance, evoked response potential (ERP) data suggests that cortical activity exhibits different voltage profiles across time depending on the engagement of separate neural/cognitive mechanisms. A peak of negative voltage at 400 ms (i.e., the “N400”) after visual presentation of a letter string indicates recognition that the letter string is pronounceable (e.g., Rossi et al., 2011). Whereas absorption/emission of light at 5.1 × 10^14^ Hz was the astronomers' first glimpse of helium, perhaps N400s at (400 ms^−1^=) 2.5 Hz is a glimpse of a similarly elemental mechanism in cognitive processes. However, neuroscience focuses its spectroscopic strategies on molecular details of blood flow and metabolites (Minati et al., 2007; Murkin and Arango, 2009). However, these molecular details alone don't address flexible, task-sensitive operation of cognitive processes of language comprehension (White et al., 2012). So long as these mechanisms are known by their characteristic time scales, why hasn't neuroscience situated the N400 on a spectrum too?

One obstacle is that spectroscopy needs long, densely sampled time series. Any single stream of ERP data is so noisy that observing N400s in single-participant data requires averaging over at least 45 trials (e.g., Niedeggen et al., 1999). Otherwise, we might collect prolonged series of ERP data of a participant viewing continuous text of pronounceable letter strings. Reading pace is ~250 ms/word (Rayner and Clifton, 2009). Let us imagine the resulting ERP signal: N400 peaks for each string, spaced 250 ms apart over time. The emission line in power-spectral analysis of this ERP signal would appear at (250 ms^−1^=) 4 Hz. Dyslexic readers take 500 ms/word longer (Russeler et al., 2007), and their N400 peaks might be spaced by (250 + 500=) 750 ms, producing a peak in a spectrum of ERP data at (750 ms^−1^=) 1.33 Hz. Just as a peak voltage at 400 ms might signify a phonotactic mechanism's characteristic scale, the gap between 1.33 and 4 Hz should indicate the difference in reading mechanisms between dyslexic and typical readers. After all, wasn't it a similar spectral difference that helped astronomers distinguish helium from sodium?

Results from reading reaction times tell a different story. Over the course of reading a 14000-word story, reading time per word decrease according to Newell and Rosenbloom's (1981) ubiquitous power-law of learning (Wallot et al., 2013). Also, rather than looking at the power spectrum of ERP signals, we might examine the power spectrum of trial-by-trial reading times. Whereas our above ERP series are imaginary, the latter power spectra have been empirically recorded and presented many times over (e.g., Van Orden et al., 2003; Holden et al., 2009; Wallot and Van Orden, 2011). These spectra show that fluctuations in reading-time series resemble 1/f noise, an inverse power-law relationship between oscillatory power and frequency. Rather than having cleanly individuated peaks like emission spectra, the power spectra from these reading-time series show a continuous slow decrease in oscillatory power with greater frequencies. Rather than individuated peaks (i.e., characteristic time scales), these spectra show similar decreases in power across all scales. Often hotly contested as statistical artifacts of “simpler” behavior of cognitive processes at characteristic scales, these patterns have survived statistical rigors (Delignières and Marmelat, 2012).

Statistical rigor notwithstanding, origins and relevance of fractal patterns in neuroscience remain as hotly contested. My own view aligns with one expressed in astronomical literature: fractal patterns reflect cascade dynamics both supported by and giving rise to structures at many scales (Larson, 2005). Astronomy and neuroscience alike have grappled with the realization that structures must somehow embody stability but also flexibility. Stars are not static, homogeneous objects distinct from their contexts—no matter the convenience of this notion for brief measurement and modeling. Stars condense out of clouds, undergo developmental phases, and collapse or explode, and so on. Structures exhibiting characteristic scales demand reconciliation with the fractal patterns inherited from the Big Bang (Mohaved et al., 2011). Similarly, independent mechanisms underpinning cognition are no more static or distinct. Brain structures and cognitive structures reflect relatively stable configurations of neural dynamics within contexts structured at multiple scales (Buzaki, 2006). They exhibit relatively stable short-range functions, but this stability is relative to longer-term variation across the time scales of learning, the life span, and species evolution. The hierarchical nesting of these multiple scales engenders cascades giving rise to structure, and these cascades are no less valid a factor in a physicochemical account than electron configurations. In this light, fractal results that can be (rigorously!) demonstrated to reflect cascade dynamics support a physicochemical account of structure, in astronomy and neuroscience alike.

Spectroscopic work relating fractal patterns to changes in the organization of observed structures supports the foregoing proposals. Fractal modeling of cloud dispersion predicts galactic emission spectra (Bottorff and Ferland, 2001) as well as temperature changes associated with star formation (Pan and Padoan, 2009). In cognitive tasks, bodily movements (e.g., of eye-gaze, hand, foot, or posture) incident to exploring task environments exhibit fractal power spectra. These power-law exponents describing these spectra serve to predict the flexibility of cognitive performance in the same tasks. That is, fractal fluctuations in the human body support the ability of cognitive systems to fine-tune their perceptual judgments (Stephen and Hajnal, 2011; Palatinus et al., 2013) or to discover new representations of problem-solving tasks (Stephen and Dixon, 2009; Stephen et al., 2009). Moreover, these effects of fractal patterning in exploratory behaviors may predict individual-trial performance above and beyond average differences in reaction times due to traditional cognitive processes (Stephen and Anastas, 2011).

The central appeal of fractal results in cognition and neuroscience, to my view, is that they may offer us a framework for aligning physicochemical accounts of neural, cognitive phenomena with physicochemical accounts pursued in different domains. Reaching for a relatively more generic physicochemical framework in which insights from different domains might be mutually relevant and compatible interests me. Not only that, it strikes me as an ideal way of grounding our tests of physicochemical guesses for neuroscience upon stronger physicochemical foundations. Evidence of fractality in domains beyond cognition and neuroscience is a reason that neuroscientists cite for being unimpressed: for instance, the fact that many more systems are found to exhibit fractal fluctuations than are agreed upon to be “cognitive” is taken to entail that fractality is not important to cognition (Botvinick, 2012). This logic seems to presume that welcome causal players in cognitive theory include only those that maintain the (pre-theoretical) distinction between cognitive systems and non-cognitive ones. Cognitive neuroscience sometimes takes great comfort in asserting the fundamental difference of cognitive systems from all others (Wagenmakers et al., 2012).

Perhaps similarity between cognitive neuroscience and other physicochemically-oriented fields is unwelcome. I find declaring one's own scientific field to require special and different explanation from other scientific fields no more compelling than Comte (1835) found pre-spectroscopic astronomy's guesswork at dots and smears in telescope images. We already have one Big Bang from which to weave cosmological history, and the simple assertion that cognitive systems are fundamentally different from everything else post-Big Bang will require another. Any such cognitive Big Bang (e.g., “when something might have had the first thought”) seems less like compelling explanation and more like reluctance to face what may be humbling physicochemical realities. I remain cautiously confident that spectroscopy should be as valuable to cognitive neuroscience as it has been to astronomy in discerning common explanatory ground with other physicochemical disciplines.

Fractal and non-fractal results from spectroscopy appear important to me because they make falsifiable the interesting physicochemical hypothesis that development of structure in nervous systems depends on cascades. When this hypothesis fails to be interesting, I will oblige my critics and stop worrying about fractals.
